# Functionalized magnetic nanoparticles remove donor-specific antibodies (DSA) from patient blood in a first ex vivo proof of principle study

**DOI:** 10.1038/s41598-024-66876-3

**Published:** 2024-07-09

**Authors:** Francis Lauener, Martin Schläpfer, Thomas F. Mueller, Seraina Von Moos, Stefanie Janker, Simon Doswald, Wendelin J. Stark, Beatrice Beck-Schimmer

**Affiliations:** 1 Institute of Anesthesiology, University Hospital Zurich (USZ), University of Zurich (UZH), 8001 Zurich, Switzerland; 2 Institute of Physiology, University of Zurich (UZH), 8057 Zurich, Switzerland; 3 Department of Nephrology, University Hospital Zurich (USZ), University of Zurich (UZH), 8001 Zurich, Switzerland; 4 Functional Materials Laboratory, Swiss Federal Institute of Technology (ETH) Zurich, 8049 Zurich, Switzerland

**Keywords:** Nephrology, Nanoparticles

## Abstract

The presence of donor-specific antibodies (DSA) such as antibodies directed against donor class I human leucocyte antigen (e.g., HLA-A) is a major barrier to kidney transplant success. As a proof of concept, functionalized magnetic nanoparticles have been designed to eliminate DSA from saline, blood and plasma of healthy donors and sensitized patients. Specific HLA-A1 protein was covalently bound to functionalized cobalt nanoparticles (fNP), human serum albumin (HSA) as control. fNP were added to anti-HLA class I-spiked saline, spiked volunteers’ whole blood, and to whole blood and plasma of sensitized patients ex vivo*.* Anti-HLA-A1 antibody levels were determined with Luminex technology. Antibodies' median fluorescent intensity (MFI) was defined as the primary outcome. Furthermore, the impact of fNP treatment on blood coagulation and cellular uptake was determined. Treatment with fNP reduced MFI by 97 ± 2% and by 94 ± 4% (*p* < 0.001 and *p* = 0.001) in spiked saline and whole blood, respectively. In six known sensitized anti-HLA-A1 positive patients, a reduction of 65 ± 26% (*p* = 0.002) in plasma and 65 ± 33% (*p* = 0.012) in whole blood was achieved. No impact on coagulation was observed. A minimal number of nanoparticles was detected in peripheral mononuclear blood cells. The study demonstrates—in a first step—the feasibility of anti-HLA antibody removal using fNP. These pilot data might pave the way for a new personalized DSA removal technology in the future.

## Introduction

Kidney transplantation improves quality of life and decreases mortality compared to dialysis^1^. Antibody-mediated rejection (AMR) due to donor-specific antibodies (DSA) is a main reason for long-term allograft failure^2^. These antibodies are directed against the human leucocyte antigens (HLA) of the donor. DSA removal could be a highly specific and less toxic treatment strategy^3^ for AMR. Additionally, it could facilitate finding a living or deceased donor for sensitized patients desperately waiting for an organ^4^. Moreover, with the elimination of DSA, waiting time could be significantly reduced for these patients.

Until now, there are no clear treatment recommendations for acute and especially chronic AMR^5^. The current multitude of therapeutic options, for example plasmapheresis, immunoadsorption, intravenous immunoglobulin, proteasome inhibitors (e.g., bortezomib), antithymocyte globulin, complement inhibitors or anti-B-cell agents (e.g., rituximab)^6–9^, lack efficacy. More recently, newer agents such as imlifidase, clazakinumab, and daratumumab offer some promise. Nevertheless, plasmapheresis and immunoadsorption are still the main pillars of reducing DSA by antibody removal in AMR or desensitization^8,10,11^. However, both methods are unspecific. Plasmapheresis removes rather unselectively valuable blood components, immunoadsorption largely IgG-immunoglobulins. Moreover, plasmapheresis can cause minor events (rash, itching, flushing, tachycardia, headache, nausea, shortness of breath, paresthesia, and hypotension or hypertension) in up to 10.9% and major adverse events such as anaphylaxis with hypotension and airway edema in up to 1.4% of patients^11^. Hence, more efficient and safe treatment strategies for AMR are needed.

**Figure 1:**
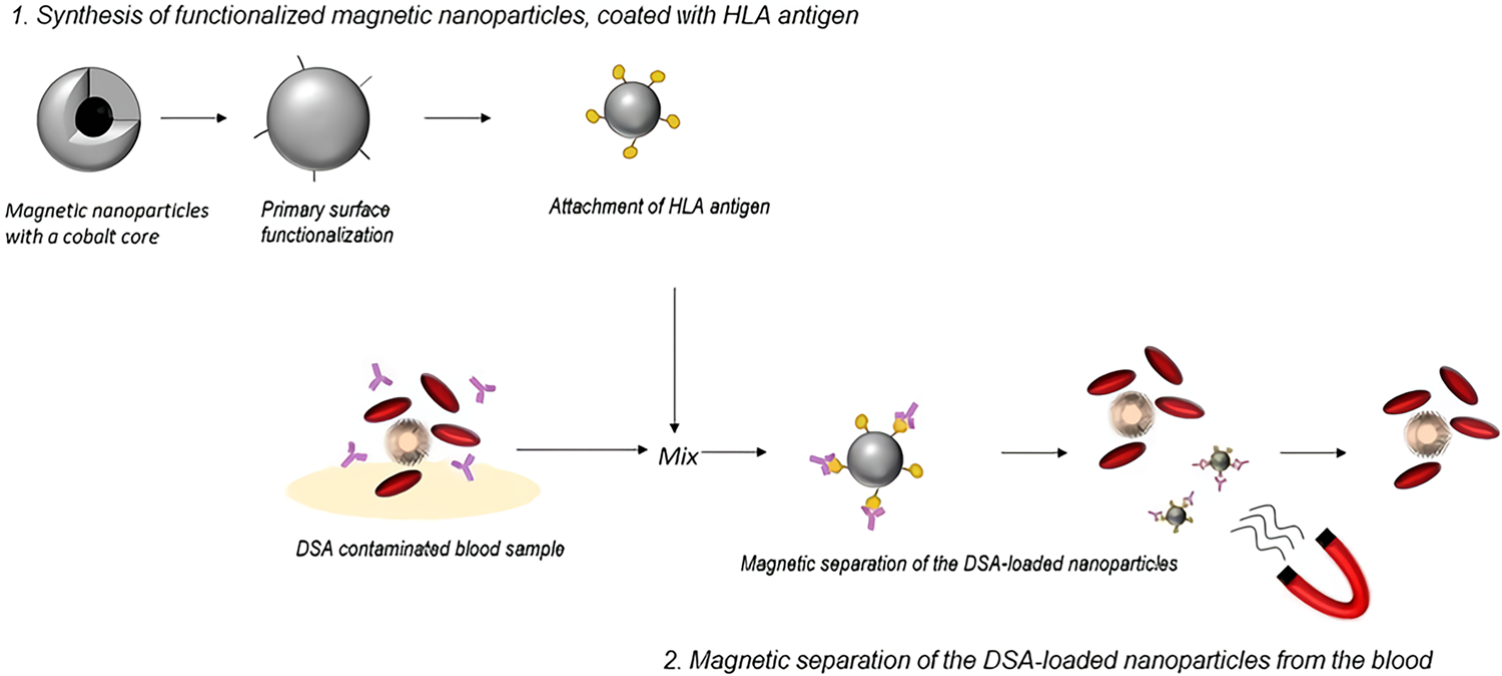
Adapted from Herrmann IK et al.^16^. A specific HLA antigen, the target of the DSA, is bound to functionalized magnetic nanoparticles. The nanoparticles are then added to blood, bind to their target, and are removed using a magnetic field. This concept is the prototype of an extracorporeal blood purification system. DSA: donor specific antibodies, HLA: human leucocyte antigen.

The aim of this study was a first preclinical proof of concept to test DSA removal by a new method using functionalized nanoparticle (fNP). Our research group has successfully established protocols for the production of carbon-coated iron as well as cobalt magnetic nanoparticles for the elimination of metal ions (Pb2+)^12^, steroid drugs (digoxin)^12–14^, inflammatory proteins (interleukin-1β, interleukin-6)^13,14^, and cancer cells from blood^15^. The developed nanoparticles are highly mobile, have an ultra-strong magnetic binding, an exceptionally high surface area, and a large binding capacity^14^. A nanoparticle-based approach for extracorporeal DSA removal, as illustrated in Fig. 1, is proposed, which could be a promising and low-side effect treatment option.

We hypothesized that it would be possible to develop nanoparticles allowing for anti-HLA antibody removal from blood, focusing on antibodies directed against class I human leucocyte antigen such as HLA-A1, without impacting blood coagulation and without major uptake of the particles by blood cells. For this approach, fNP were coated with HLA-A1 antigens (later mentioned as HLA-A1-fNP) or with human serum albumin (HSA) as negative control (HSA-fNP). A stepwise procedure was designed in which a commercially available anti-human HLA was eliminated from phosphate-buffered saline (PBS) and from volunteer blood. Thereafter, anti-HLA antibodies were removed from patients’ plasma and whole blood. Measurements were performed using median fluorescent intensity (MFI) as outcome parameter as practiced with patients’ samples determining DSA.

## Results

### Anti-HLA class I antibody removal from antibody-spiked PBS

Overall, HLA-A1-fNP treatment reduced MFI from spiked PBS by 97 ± 2% (*p* < 0.001) while a reduction of 27 ± 8% (*p* = 0.024) was observed with HSA-fNP. MFI reduction of the individual HLA-A1-fNP batches (b1, b2, and b3) and the corresponding HSA-fNP batches (c1, c2, and c3) are presented in Table 1 with previous testing of various nanoparticle concentration (supplementary Table 1).

**Table 1:** Relative and absolute MFI reductions in antibody-spiked PBS and whole blood by treatment with HLA-A1-fNP and HSA-fNP.

fNP type	Batch designation	Relative MFI reduction	Absolute MFI reduction	Absolute pre-treatment MFI	Absolute post-treatment MFI
MFI reduction in antibody-spiked PBS (n=3)					
HLA-A1-fNP	b1	96%	9688	10,134	446
HLA-A1-fNP	b2	96%	2984	3120	136
HLA-A1-fNP	b3	99%	7377	7473	96
Mean MFI reduction ± SD	–	97 ± 2% (*p* < 0.001)	–	–	–
HSA-fNP	c1	20%	1120	5676	4556
HSA-fNP	c2	35%	2031	5856	3825
HSA-fNP	c3	27%	2012	7473	5461
Mean MFI reduction ± SD	–	27 ± 8% (*p* = 0.024)	–	–	–
MFI reduction in antibody-spiked whole blood of volunteers (n=3)					
HLA-A1-fNP	b1	97%	5599	5751	152
HLA-A1-fNP	b2	89%	9138	10,266	1128
HLA-A1-fNP	b3	96%	8985	9351	366
Mean MFI reduction ± SD	–	94 ± 4% (*p* = 0.001)	–	–	–
HSA-fNP	c1	8%	1036	13,618	12,582
HSA-fNP	c2	4%	448	10,266	9818
HSA-fNP	c3	-2%	− 159	9351	9510
Mean MFI reduction ± SD	–	3 ± 5% (*p* = 0.375)	–	–	–

The antibody concentration was 5 μg/ml, HLA-A1- and HSA-fNP concentration was 0.8 μg/μl. For each fNP-type, three batches were produced to test reproducibility. The “-” indicates an increase in MFI. Pre-treatment corresponds to the MFI before the sample was treated with fNP, post-treatment to the MFI after fNP treatment. MFI: median fluorescence intensity, PBS: phosphate-buffered saline, HLA: human leucocyte antigen, HSA: human serum albumin, fNP: functionalized nanoparticles, b1-3: HLA-A1-fNP batch 1–3, c1-3: HSA-fNP batch 1–3, SD: standard deviation.

### Anti-HLA class I antibody removal from antibody-spiked volunteer whole blood

HLA-A1-fNP treatment reduced MFI from spiked whole blood by 94 ± 4% (*p* = 0.001), treatment with control HSA-fNP by 3 ± 5% (*p* = 0.370). MFI reduction of the individual batches is presented in Table 1.

### Anti-HLA-A1 antibody removal from patient samples

#### Study population

Between 14/05/2021 and 20/10/2021, eleven patients were recruited with previously known titer of DSA. Blood samples of five patients had to be excluded because they were anti-HLA-A1 antibody negative at the time of study inclusion. Six blood samples were used for the final analysis. The patient flow is presented in supplementary Fig. 1.

### Anti-HLA-A1 antibody removal from patient plasma

In the plasma of all 6 included patients (supplementary Fig. 1), HLA-A1-fNP treatment reduced MFI. The average (relative) reduction was 65 ± 26% (*p* = 0.002). Treatment with HSA-fNP did not impact MFI values. Details of the achieved MFI reduction using optimized HLA-A1-fNP concentrations are presented in Table 2. In four patients, HLA-A1-fNP concentration established in the spiked plasma samples had to be increased to achieve considerable MFI reduction (patient 1 to 1.2 µg/µl, patient 10 to 1.8 µg/µl, and patients 2 and 4 to 2.3 µg/µl), while for all tests the concentration of HLA-A1-fNP was 0.8 μg/μl.

**Table 2:** Relative and absolute maximum reduction values in patient plasma and patient whole blood.

Patient	Relative MFI reduction	Absolute MFI reduction	Absolute pre-treatment MFI	Absolute post-treatment MFI
Maximum MFI reduction in patient plasma (n=6)				
1	96%	4729	4940	211
2	31%	1359	4427	3068
4	83%	7826	9400	1574
6	78%	9639	12,346	2707
9	39%	722	1848	1126
10	65%	5372	8291	2919
Mean MFI reduction ± SD	65 ± 26% (*p* = 0.002)	–	–	–
Maximum MFI reduction in patient whole blood (n=5)				
1	NA	NA	NA	NA
2	7%	179	2466	2287
4	84%	6673	7923	1250
6	89%	10,372	11,630	1258
9	76%	855	1126	271
10	70%	4350	6197	1847
Mean MFI reduction ± SD	65 ± 33% (*p* = 0.012)	–	–	–

Relative and absolute values for the treatment with HLA-A1-fNP in individualized doses for each patient are shown. For patient one, no whole blood was available. For the first experiment 0.8 μg/μl nanoparticles were used in plasma or whole blood, respectively. In case no relevant MFI reduction was observed, higher and lower concentrations were tested. Pre-treatment corresponds to the MFI before the sample was treated with fNP, post-treatment to the MFI after fNP treatment. MFI :  median fluorescence intensity, HLA:  human leucocyte antigen, fNP:  functionalized nanoparticles, NA: not available, SD:  standard deviation.

### Anti-HLA-A1 antibody removal from patient whole blood

Average MFI reduction upon HLA-A1-fNP treatment was 65 ± 33% (*p* = 0.012). HSA-fNP treatment did not impact MFI values. Details of the achieved MFI reduction using optimized HLA-A1-fNP concentrations are presented in Table 2. For one patient (patient 1), no whole blood was available.

In three patients (patients 2, 4, and 10) HLA-A1-fNP concentrations had to be increased to achieve MFI reduction.

### Specificity of the HLA-A1-fNP

As known from the analyses performed in the hospital all patients included in the patient study were positive for several HLA class I antigens. We therefore tested in the plasma and whole blood of the six patients how specific HLA-A1-fNP treatment was in reducing also other DSA within the HLA class I. As presented in Table 3 several HLA class I antigens such as A3, A11:01, A11:02, A24:03, A29:02, A30:02, A36, A68:01, A68:02, A80, B44:02, B44:03, B45, and B76 were removed in varying amounts (MFI reduction of 17% to 85%), but also varying from patient to patient. The average reduction and the number of patients is indicated in Table 3, supplementary Tables 2 and 3. Exemplary in one patient, the raw MFI values over the whole tested HLA class I antigen panel before and after treatment with HLA-A1-fNP of whole blood and plasma are shown in supplementary Figs. 2 and 3.

**Table 3:** Average relative MFI reduction in plasma and whole blood for anti-HLA class I antibodies from all 6 patients with a reduction > 30%.

	HLA class I antigen														
	A1	A3	A11:01	A11:02	A24:03	A29:02	A30:02	A36	A68:01	A68:02	A80	B44:02	B44:03	B45	B76
Plasma	65 ± 26%, n = 6	35%, n = 1	96%, n = 1	97%,n = 1	86%, n = 1	49%, n = 1	17%*, n = 1	75 ± 29%, n = 3	92%, n = 1	85%, n = 1	60 ± 23%, n = 2	64%, n = 1	59%, n = 1	76 ± 6%, n = 2	63 ± 14%, n = 2
Whole blood	65 ± 33%, n = 5	70 ± 33%, n = 2	99%, n = 1	67 ± 45% n = 2	88%, n = 1	58%, n = 1	32%, n = 1	71 ± 29%, n = 2	79 ± 11%, n = 2	59%, n = 1	68 ± 11%, n = 2	63%, n = 1	69%, n = 1	65 ± 16%, n = 2	64 ± 13%, n = 2

For comparability, the MFI reduction of HLA-A30 in plasma is also reported (marked with an *), although it was not reduced > 30% in plasma. For some antigens, there is more than one allele available in the Luminex multiplex assay. This is indicated by the second number after the colon. MFI:  median fluorescence intensity, HLA : human leukocyte antigen.

Supplementary Table 4 shows the much lower relative MFI reduction of HLA class II antibodies with values between 5 and 18%, which were determined in the plasma of patient number 1.

### Secondary outcomes

#### Coagulation analysis by rotational thromboelastometry

**Figure 2:**
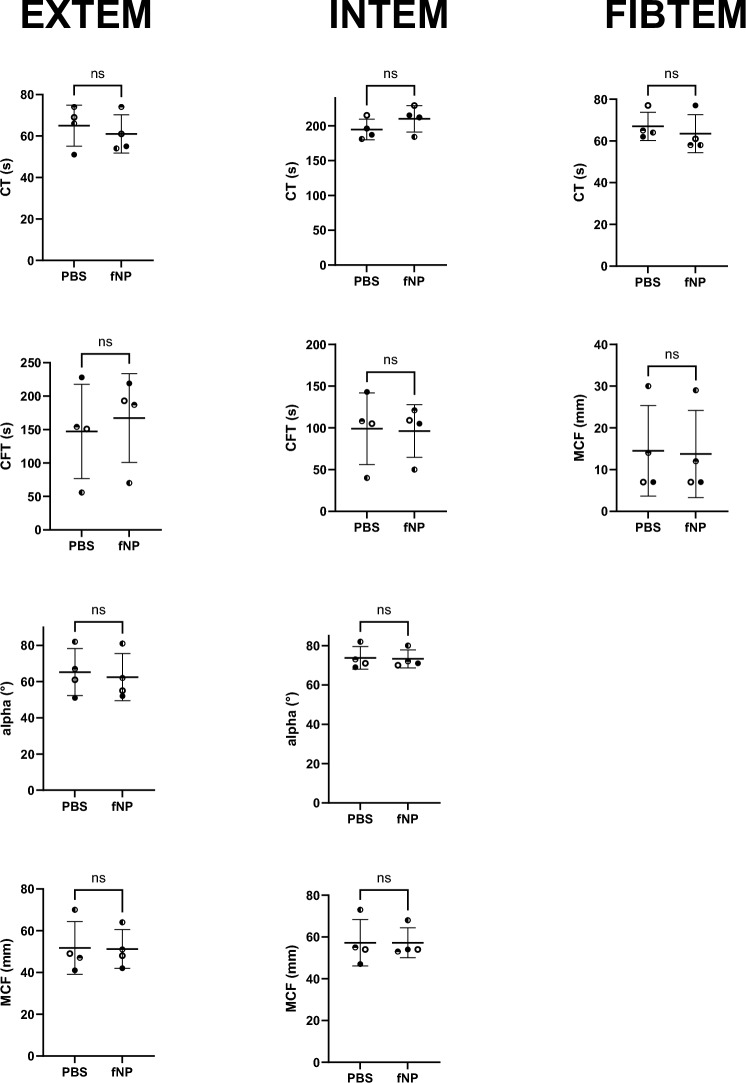
For EXTEM, INTEM, and FIBTEM, every parameter is depicted in one plot. Individual values, mean, and standard deviations are depicted. Each symbol represents one patient (n = 4). CT: clotting time, CFT: clot formation time, MCF: maximum clot formation, fNP: functionalized nanoparticles, PBS: phosphate-buffered saline, ns: non-significant. The reference values are: EXTEM: CT 38–79 s, CFT 34–159 s, alpha-angle 63–183°, MCF 50–72 mm; INTEM: CT 100–240 s, CFT 30–110 s, alpha-angle 70–83°, MCF 50–72 mm; FIBTEM: CT 38–62 s, MCF 8–24 mm.

As nanoparticles with their large surface could potentially interact with the coagulation system and enhance coagulation leading to thrombosis or inhibit coagulation, increasing the risk of bleeding, a coagulation analysis was performed. ROTEM® was used to determine two coagulation pathways, namely EXTEM (extrinsic coagulation pathway) and INTEM (intrinsic pathway), as well as fibrin polymerization (FIBTEM). In all ROTEM® tests (i.e. EXTEM, INTEM, FIBTEM), there was no significant difference in any of the parameters, i.e. clotting time (CT), clot formation time (CFT), alpha angle (α) and maximum clot firmness (MCF) between HSA-fNP and PBS treated samples (Fig. 2).

### Cellular uptake of fNP

**Figure 3:**
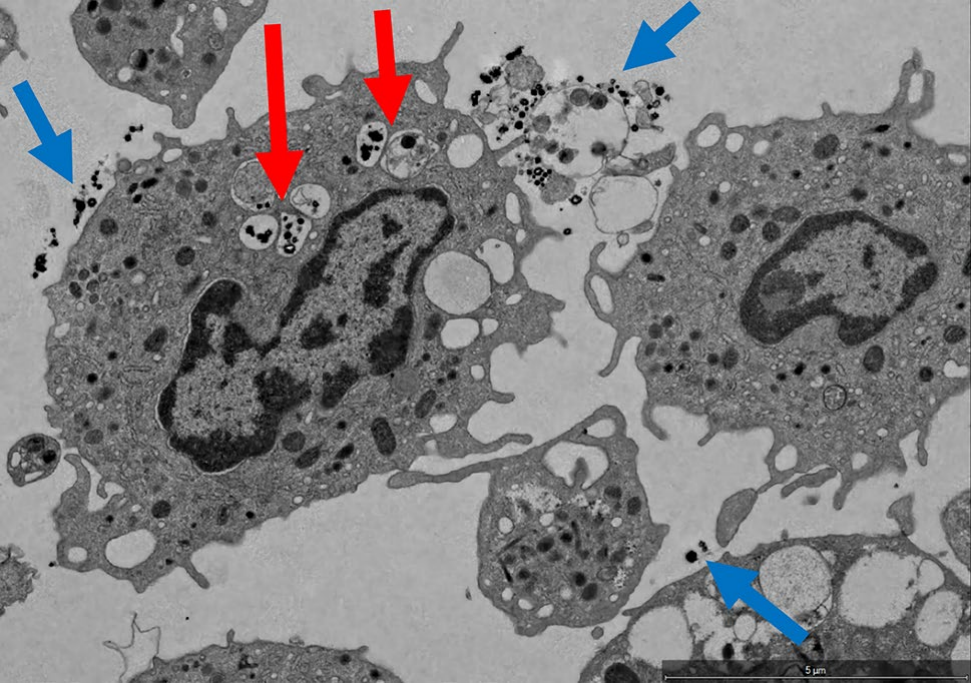
Transmission electron microscopy (TEM) scans of PBMC. fNP are black structures within vacuoles, this was confirmed by EDX analysis. EDX-detected fNP are indicated with a red arrow. Extracellular fNP are present in this scan (blue arrows). TEM: transmission electron microscopy, PBMC: peripheral blood mononuclear cells, EDX: energy dispersive X-ray spectroscopy, fNP: functionalized nanoparticles.

Cellular uptake has been described when using nanoparticles, and therefore a risk assessment was considered essential focusing on a static condition such as observed in capillaries with a slow blood flow as well as on a dynamic condition, found in peripheral veins. Incubation of patient whole blood (n = 4) with fNP under static conditions resulted in fNP uptake in PBMC in three of four samples. Overall, 6 of 467 cells (1.28%) were fNP positive. fNP were mainly located in vacuoles in cytoplasmic areas. In a volunteer whole blood sample, no fNP positive cells were found. Figure 3 illustrates an fNP positive PBMC. More detailed information about fNP uptake into PBMC are provided in Table 4.

**Table 4:** informs about the number of fNP positivity in four patients and one healthy volunteer, how many PBMC have been found and the percentage of nanoparticle-positive cells in transmission electron microscopy scans upon fNP exposure under static conditions. fNP = functionalized nanoparticles, PBMC = peripheral blood mononuclear cells.

Sample name	Number of PBMC containing fNP	Total number of PBMC	Percentage of PBMC containing fNP (%)
Patient 2	2	89	2.25
Patient 4	3	126	2.38
Patient 10	0	48	0
Patient 11	1	204	0.49
Volunteer	0	367	0

Flowing conditions (supplementary Figs. 4, 5) decreased fNP uptake. Four of 11,693 cells (0.0003%) were fNP positive in 9 blood samples from one volunteer. Details about all measurement time points, the number of fNP positive PBMC, the total number of PBMC and the percentage of fNP positive PBMC are indicated in Table 5.

**Table 5:** indicates the number of fNP positivity in nine blood samples from volunteers (S = sample), how many PBMC have been found and the percentage of nanoparticle-positive cells in transmission electron microscopy scans upon fNP exposure under flowing conditions. fNP = functionalized nanoparticles, PBMC = peripheral blood mononuclear cells.

	Sample name	Number of PBMC containing fNP	Total number of PBMC	Percentage of PBMC containing fNP (%)
Incubation time: 30 s	S1	0	462	0
	S2	2	1136	0.18
	S3	1	1148	0.09
Incubation time: 120 s	S4	0	2301	0
	S5	1	1041	0.10
	S6	0	1589	0
Incubation time: 300 s	S7	0	1046	0
	S8	0	683	0
	S9	0	2287	0

## Discussion

Previous research has used antibodies attached to nanoparticles to extract molecules like digoxin and interleukin-1ß^13^ and even circulating tumor cells^17^. However, a targeted antibody removal from blood using functionalized nanoparticles with an attached antigen has not been performed until now. Our preclinical experiments confirm the feasibility of anti-HLA antibody removal in a simple (PBS) and a more complex environment (whole blood) with HLA-A1-fNP nanoparticles.

Testing functionality of the antigen bound to the particles is demonstrated by a 97% MFI reduction, which was significantly higher than the observed 35% reduction when using control (HSA-fNP) particles. These results are in line with previous data showing specific removal of items by fNP and a limited unspecific reduction by control fNP^15,17^, which might be caused by non-specific protein–protein interactions^18^.

Not surprisingly the removal quantity of anti-HLA antibodies from patient plasma in our small cohort varied widely. This is due to polyclonality and differences in binding specificity^19^, to the large differences of anti-HLA antibody titers in the individual patients (MFIs ranging from 1848 to 12,346), to a known inevitable inaccuracy of MFI-measurements by Luminex® bead technology and to cross-reactivity^20^. Such cross-reactive antibodies are not targeted against HLA-A1 but may interact with HLA-A1 with weaker affinity, therefore apparently decreasing removal efficiency and, at the same time, specificity. In theory, cross-reactive antibodies may lead to early saturation of fNP. Such issues could be addressed by increasing the nanoparticle concentration. Of note, excessively high levels of HLA antibodies can inhibit binding due to steric effects (known as the hook effect)^21,22^ and thereby compromise antibody removal. Another reason for incomplete anti-HLA-A1 antibody removal might be the presence of IgA and IgM antibodies targeted to HLA-A1 which could compete for the HLA-binding site on the fNP^21^. The occupied binding site prevents binding of IgG, the only antibody type detected by the Luminex® assay. To tackle this issue, again the fNP concentration could be adapted for a therapeutic approach, according to the patient’s antibody concentration.

There are additional potential interferences with the Luminex method. While rather unlikely in our study, they will be briefly discussed for completeness. Medications such as polyclonal anti-thymocyte globulin preparations (e.g., thymoglobulin) may interfere with HLA antibody detection tests^21,23^. Anti-thymocyte globulin is typically administered during renal transplantation^24^. While four of the study participants had received anti-thymocyte globulin during their transplant, an interaction is unlikely because of drug administration years before enrolment in our study. Intravenous immunoglobulins are another type of drug that may contain human HLA antibodies^25^. Only one patient had received immunoglobulins. A relevant effect can be excluded as the administration happened five years before study inclusion.

HLA-A1-fNPs substantially reduced other anti-HLA class I antibodies. Most of these antibodies target structurally similar epitopes^20,26–28^. Previously, it has been reported that there is cross-reactivity between all the antibodies removed in this study, except for anti-HLA-B44 and -45 antibodies^20,28^. Nevertheless, cross-reactivity for B44 and B45 is conceivable because they share two common eplets with HLA-A1, namely 99Y and 193 PI^26^. This removal of other HLA-class I antibodies with antigenic mimicry might also be therapeutically important as these antibodies likely also mitigate negative anti-donor allograft immune responses. However, eplet mapping with recognition of shared epitopes by different antibody specificities could help explain the reduction of other than anti-HLA A1 antibodies. In a future study, data retrieved by Luminex® should be verified using another platform such as Immucor (Immucor Medizinische Diagnostik GmbH, Dreieich, Germany).

When considering fNP in a therapeutic approach, safety aspects such as impact on blood coagulation are crucial as nanoparticles have been shown to impact coagulation before functionalization^29,30^. In a previous study, functionalized nanoparticles as those used in this trial did not impact coagulation^29^. This was also confirmed in the current study by thromboelastometry, which showed normal values.

An additional potential danger in using nanoparticles is the uptake by phagocytic cells with unknown long-term effects of intracellular nanoparticles. The surface layer of nanoparticles, also known as the “corona”, appears to determine toxicity^31^. Nanoparticles with a hyperbranched polyglycerol layer, as used in these experiments, are considered biocompatible and have low tissue toxicity^32,33^. Several factors impact cellular nanoparticle uptake into various cells such as electrostatic interactions^34^, shear stress^35,36^, elasticity^37^, and shape^38^ of nanoparticles. In previous studies, we found only a small fraction of particles which are taken up by phagocytic cells^29^. Experiments presented here complement previous knowledge: short exposure time and flow conditions minimize nanoparticle uptake^39^.

Our study has some limitations. The sample size of sensitized patients was small, and testing was done only for one antigen (HLA-A1). In addition, the known cross-reactivity and measurement inaccuracy of the Luminex® assay interferes with the analysis of the efficiency in antibody removal by the nanobeads. However, as Luminex® is the most widely used technology the results allow a direct translation into the routine clinical scenario. Our pilot results realistically support the feasibility of designing even epitope-specific magnetic nanobeads in the future. The test results in healthy volunteers and patients, in plasma and whole blood indicate that fNP production is both effective in the removal of antibodies with minimal side effects. A limitation of the application of fNP in patients is the potential deposition of nanoparticles. Intracellular nanoparticles were detected semi-quantitatively by electron microscopy. For clinical therapies, absolute quantification of the nanoparticle amount and the rate of release of cobalt ions into the blood will have to be determined. Moreover, fNP stability will have to be confirmed: the release of HLA from particles or phagocytic uptake of HLA bound to fNP’s might—at least technically—boost sensitization. Despite these limitations, removing DSA using fNP technology is possible and has potential as a personalized therapy to combat graft rejection in affected patients. Most importantly, these limitations should be addressed in future studies, and the overall low side-effect profile of this technology must be set into perspective with the impact of DSA and immunosuppression on patient health and transplant outcomes.

In summary, this proof of principle study shows for the first time that removal of DSA is possible via custom-made specific magnetic nanobeads. This might open the possibility of targeted desensitization in organ transplantation, facilitating organ allocation, reducing waiting times, and prolonging transplant survival.

## Materials and methods

### Ethical aspects

The study was conducted at the University Hospital Zurich, Switzerland and in accordance with good laboratory practice, the Declaration of Helsinki, as well as legal and institutional standards. Ethical approval from the cantonal ethics committee (Kantonale Ethikkommission, Stampfenbachstrasse 121, CH-8090 Zurich, Switzerland) was obtained for volunteers (CEC 2016–01140, 2016/11/21) and for patients (CEC 2020–00295, 2020/04/02). Informed consent was obtained from all subjects and/or their legal guardian(s). The consent form was signed by volunteers and patients before blood was drawn. Inclusion criteria were age ≥ 18 years and no known major diseases for healthy volunteers. For patients, inclusion criteria were age of ≥ 18 years, and positive anti-HLA-A1 antibodies with a MFI > 1000.

### Nanoparticle synthesis and functionalization

Carbon-coated cobalt nanoparticles were synthesized by reducing flame spray synthesis with the addition of acetylene to the nanoparticle-forming flame, as described previously^40^. Manufactured particles are highly magnetic with a saturation magnetization of 158 A m^2^ kg^-1^^40^. The outermost carbon layer was covalently functionalized with amino phenethyl alcohol by diazotization^40^ and subsequently coated with hyperbranched polyglycerols by a polymerization reaction adapted from Daniel Wilms and colleagues^32^. Subsequently, the coated nanoparticles were functionalized with succinic anhydride to form carboxylic acid end-groups for the subsequent conjugation, adapted from Yang and colleagues^41^. A class I human leucocyte antigen (HLA-A*01:01, Pure Protein LLC, Oklahoma City, USA, catalog number A0101) was covalently bound to the polymer. The protein is a recombinant, truncated, naturally folded, glycosylated HLA-A1 protein, which consists of all five subunits (alpha1, alpha2, alpha3, beta-2-microglobulin, and an endogenous peptide). A primary amine of the protein was linked to a carboxylic acid group of the hyperbranched polyglycerol chains to form a stable amide bond by carbodiimide conjugation with 1-ethyl-3-(-3-dimethylaminopropyl) carbodiimide (EDC) (Thermo Fisher Scientific, Waltham, MA, USA) and N-hydroxysuccinimide (NHS) (Sigma-Aldrich, St. Louis, MO, USA, catalog number 130672). As a negative control, human serum albumin (HSA) (Sigma-Aldrich, St. Louis, MO, USA, catalog number H4522) was linked to the nanoparticles instead of the HLA-A1 protein. For 1 mg of nanoparticles, 60 μg of protein (HLA-A1 or HSA) was used.

### Antibody removal with functionalized nanoparticles

#### Anti-HLA class I antibody removal from antibody-spiked PBS

PBS was spiked with 5 μg/ml of a W6/32 mouse monoclonal anti-human HLA ABC antibody (clone BE0079 [HB-95], kindly provided by Dr. Rico Buchli, Pure MHC, Oklahoma City, USA, lot number L717820A2). The concentration was determined through serial dilution testing to achieve a MFI for HLA-A1 within a range of 1000–10,000. fNP were sonicated in iced water (Bandelin SONOREX™ Digital 10 P Ultrasonic bath, Merck®, Darmstadt, Germany) five times for one minute at the lowest intensity (12-Watt nominal power at 35kHz) with a one-minute break after each cycle. In each break, fNP were vortexed briefly.

After testing concentrations of 0.5 and 1.2 μg/μl, the following experiments were performed with a fNP concentration of 0.8 μg/μl in the antibody-spiked PBS samples (supplementary Table 1). Antibody-spiked PBS was incubated with fNP (five minutes on a rocker; RM5, M. Zipperer GmbH, Dottingen, Germany), followed by a removal of the fNP by a neodymium magnet (1cm^3^, *B* = 1.4 T). Within 10 s, fNP, directed by the magnet, formed a visible layer on the tube wall. The purified sample was carefully transferred to a fresh tube. Touching the nanoparticle layer during sample transfer was avoided.

### Anti-HLA class I antibody removal from antibody-spiked volunteer whole blood

Citrate whole blood from healthy volunteers with consent was spiked with 5 μg/ml W6/32 mouse monoclonal anti-HLA ABC antibody. fNP removal was performed as described. Prior to antibody detection by Luminex® assay performed in 96-well v-bottom microplates (chimney wells, Greiner Bio-One Company, Austria, catalog number 651201) whole blood samples had to be centrifuged at 2500 *g* for 10 min at room temperature to obtain plasma.

### Anti-HLA class I antibody removal from patient plasma and whole blood containing HLA-A1 positive antibodies

Removal of anti-HLA-A1 antibodies in patient samples was performed as described for spiked volunteer blood. For the first experiment 0.8 μg/μl nanoparticles were used in plasma or whole blood, respectively. In case, no relevant MFI reduction was observed, higher and lower concentrations were tested. The concentration with the highest MFI reduction was reported in the results. To determine the specificity of the HLA-A1-fNP on anti-HLA class I and II antibodies, 0.8 μg/μl of HLA-A1-fNP in plasma were used. In all patient samples anti-HLA class I antibodies were measured. Anti-HLA class II antibodies were determined only in the first patient.

### Quantification of anti-HLA-A1 antibodies

Semi-quantitative anti-HLA-A1 antibodiy measurements were performed using a multiplex assay and the Luminex® 200 platform (Thermo Fisher Scientific, Waltham, MA, USA) in combination with the LABScreen™ Single Antigen Beads HLA Class I, II and negative control (One Lambda Inc., West-Hills LA, CA, USA, catalog number LS1A04, LS2A01 and LS-NC). The current clinical standard for DSA detection is the Luminex® single antigen bead (SAB) technology^5^. It measures DSA semi-quantitatively, expressed as MFI^42–44^. There is no uniform cut-off value for DSA identification, but a positive cut-off value for a specific HLA target of > 1000 has been suggested in the literature^44^. Samples were prepared according to the manufacturer’s instructions. For patient samples, a secondary (PE)-conjugated goat anti-human IgG antibody (One Lambda Inc., West-Hills LA, CA, USA, catalog number LS-AB2), for W6/32 spiked samples, a (PE)-conjugated goat anti-mouse IgG antibody (SouthernBiotech, Birmingham, AL, USA, catalog number 1030–09) was used.

### Effect of fNP on rotational thromboelastometry

The impact of fNP on blood coagulation was assessed with rotational thromboelastometry (ROTEM® delta, Tem Innovations GmbH, Munich, Germany). EXTEM (extrinsic coagulation pathway), INTEM (intrinsic pathway), and FIBTEM (fibrin polymerization) were measured. The analyzed parameters were: clotting time (CT, in seconds, for EXTEM, INTEM, FIBTEM), clot formation time (CFT, in seconds, for EXTEM, INTEM), alpha angle (α, in degrees, for EXTEM, INTEM), and maximum clot firmness (MCF, in millimeters, for EXTEM, INTEM, FIBTEM).

Patient blood was supplemented with HSA-fNP (final concentration 0.8 μg/μl, stock-solution: 2.4 μg/μl) or the corresponding PBS volume, the carrier solution of HSA-fNP. The fNP removal was performed as described above. To detect the fNP-based effect and to ensure that inter-individual differences were weighted as little as possible, samples after fNP treatment were compared with samples after PBS treatment.

### Uptake of fNP into peripheral blood mononuclear cells (PBMC)

#### Uptake of fNP under static conditions

Cellular imaging was performed at the Center for Microscopy and Image Analysis, University of Zurich, using a FEI Tecnai G2 Spirit transmission electron microscope (TEM). To reliably detect fNP, suspicious structures on the images were examined with energy dispersive X-ray spectroscopy (EDX). Citrated whole blood samples were treated with HSA-fNP as described above with an incubation time of five minutes followed by removal with the neodymium magnet. PBMC were isolated from whole blood by density gradient centrifugation with Ficoll-Paque™ Plus (GE Healthcare Bio-Science AB, Uppsala, Sweden) according to the manufacturer’s instructions. PBMC were dispensed in fixation buffer (0.2 M sodium cacodylate, glutaraldehyde 25% in H_2_O, all from Fisher Scientific International Inc., Pittsburgh, PA, USA in distilled water). All cells, examined by TEM, were counted with the software ImageJ, version 1.53k^45^ and the “cell counter” plugin^46^.

### Uptake of fNP in flowing blood

A flowing condition setup, according to supplementary Fig. 4, was assembled. Blood was pumped through a tubing system (inner diameter: 0.9 mm, flow rate: 23 ml/h) using a syringe pump (Agilia SP, Fresenius Kabi AG, Kriens, Switzerland). 5.8 ml/min fNP solution (concentration: 2.4 μg/μl) were injected into the flowing blood (Standard PHD ultra™ CP syringe pump, Harvard Apparatus, Holliston, MA, USA). The tubing was guided through a flow chamber (μ-Slide, 1-Luer, ibiTreat, ibidi®, Munich, Germany; channel: l:50 mm, w:5 mm, h:0.4 mm), submerged in an ultrasonic bath running constantly at the lowest intensity (12-Watt nominal power at 35 kHz). fNP were removed using a magnetic bead column (MS column, Miltenyi Biotec, Bergisch Gladbach, Germany).

Blood was obtained after flowing for 30, 120, and 300 s under the above-mentioned conditions. Incubation time was adapted using different tubing lengths. Small-scale mixing is largely controlled by slow molecular diffusion due to predominantly laminar flow present in microchannels (i.e. channel widths/depths ranging from a few hundred micrometers to a few millimeters)^47^. To ensure proper mixing of the two streamlines, the setup was validated according to Aubin et al.^47^ as described in the supplementary file and the supplementary Fig. 5. The uptake of fNP into PBMC was quantified as described above.

### Statistical analyses

Data are presented as mean ± standard deviation. The relative MFI reduction was calculated, the pre-treatment MFI was defined as 100%. To compare two groups, a Student’s t-test was performed. A *p*-value < 0.05 was considered significant. All statistical analyses were performed in GraphPad Prism Version 9.2.0 (GraphPad Software, San Diego, CA, USA). The number of independent experiments is indicated in the figure legends.

### Supplementary Information

Supplementary Information.

## Data Availability

Data will be fully available upon publication (martin.schlaepfer@uzh.ch).
