# Supplementary material for: Functionalized magnetic nanoparticles remove donor-specific antibodies (DSA) from patient blood in a first ex vivo proof of principle study
Source: Sci Rep. 2024 Jul 9;14:15818. doi: 10.1038/s41598-024-66876-3 (PMC11233667; doi:10.1038/s41598-024-66876-3)
Supplement: Supplementary file 1 — Supplementary Information. [file 41598_2024_66876_MOESM1_ESM.docx]

**Functionalized magnetic nanoparticles remove donor-specific antibodies (DSA) from patient blood in a first *ex vivo* proof of principle study**

Francis Lauener *^1,2^, Martin Schläpfer*^1,2^, Thomas F Mueller^3^, Seraina Von Moos^3^, Stefanie Janker^1^, Simon Doswald^4^, Wendelin J Stark^4^, Beatrice Beck-Schimmer^1,2^

* Equal contribution as the first authors

**Affiliations:**

^1^Institute of Anesthesiology, University of Zurich, University Hospital Zurich (USZ), CH-8001 Zurich, Switzerland

^2^Institute of Physiology, University of Zurich (UZH), CH-8057 Zurich, Switzerland

^3^Department of Nephrology, University of Zurich, University Hospital Zurich (USZ), CH-8001 Zurich, Switzerland

^4^Functional Materials Laboratory, Swiss Federal Institute of Technology (ETH) Zurich, CH-8049 Zurich, Switzerland

**Supplementary information**

**Supplementary tables**

**Anti-HLA class I antibody removal from spiked PBS**

***Supplementary table 1.*** *MFI reduction in antibody-spiked PBS with different amounts of HLA-A1-fNP*

| **fNP type** | **Batch designation** | **Relative MFI reduction (%)** | **Absolute MFI reduction** | **Absolute pre-treatment MFI** | **Absolute post-treatment MFI** |
| --- | --- | --- | --- | --- | --- |
| **fNP concentration: 0.5 μg/μl** | |  |  |  |  |
| HLA-A1-fNP | b1 | 95 | 9624 | 10134 | 510 |
| HLA-A1-fNP | b2 | 58 | 1825 | 3120 | 1295 |
| HLA-A1-fNP | b3 | 97 | 7229 | 7473 | 244 |
| **fNP concentration: 1.2 μg/μl** | |  |  |  |  |
| HLA-A1-fNP | b1 | 94 | 9512 | 10134 | 622 |
| HLA-A1-fNP | b2 | 99 | 3093 | 3120 | 27 |
| HLA-A1-fNP | b3 | 99 | 7421 | 7473 | 52 |

***Supplementary table 1****. Relative and absolute MFI reductions in antibody-spiked PBS (n=3) by treatment with 0.5 and 1.2* *μg/μl HLA-A1-fNP. PBS was spiked with 5 μg/ml W6/32 antibody. Also, pre- and post-treatment MFI are reported. MFI= median fluorescence intensity, PBS= phosphate-buffered saline, HLA= human leucocyte antigen, fNP= functionalized nanoparticles, b1-b3= HLA-A1-fNP batch 1-3.*

**Specificity of the fNP**

***Supplementary table 2****. Relative MFI reduction in plasma for all reduced anti-HLA class I antibodies*

|  | **HLA class I antigen** | | | | | | | | | | | |
| --- | --- | --- | --- | --- | --- | --- | --- | --- | --- | --- | --- | --- |
| **Patient** | **A1** | **A3** | **A11** | **A24** | **A29** | **A30** | **A36** | **A68** | **A80** | **B44** | **B45** | **B76** |
| **1** | 96% |  |  |  |  |  | 96% |  |  |  |  |  |
| **2** | 31% |  |  |  |  |  |  |  |  |  |  |  |
| **4** | 83% |  |  |  | 49% | 17% | 88% | 63% | 76% |  | 80% | 53% |
| **6** | 78% |  | 97% | 86% |  |  | 82% | 92% |  |  |  |  |
| **9** | 39% |  |  |  |  |  |  |  |  |  |  |  |
| **10** | 65% | 35% | 30% |  |  |  | 32% |  | 43% | 64% | 71% | 73% |

**Supplementary table 2**. Relative MFI reduction in plasma for anti-HLA class I antibodies with an initial MFI > 1000 and an MFI reduction ≥30% by treatment with fNP. For comparability with the supplementary table, the MFI reduction of HLA-A30 is also shown. MFI= median fluorescence intensity, HLA= human leukocyte antigen.

***Supplementary table 3****. Relative MFI reduction in whole blood for all reduced anti-HLA class I antibodies*

|  | **HLA class I antigen** | | | | | | | | | | | |
| --- | --- | --- | --- | --- | --- | --- | --- | --- | --- | --- | --- | --- |
| **Patient** | **A1** | **A3** | **A11** | **A24** | **A29** | **A30** | **A36** | **A68** | **A80** | **B44** | **B45** | **B76** |
| **1** | NA | | | | | | | | | | | |
| **2** | 7% |  |  |  |  |  |  |  |  |  |  |  |
| **4** | 84% |  |  |  | 58% | 32% |  | 71% | 75% |  | 54% | 54% |
| **6** | 89% | 93% | 99% | 88% |  |  | 91% | 87% |  |  |  |  |
| **9** | 76% |  |  |  |  |  |  |  |  |  |  |  |
| **10** | 70% | 46% | 43% |  |  |  | 50% |  | 60% | 69% | 76% | 73% |

**Supplementary table 3**. Relative MFI reduction in whole blood for anti HLA class I antibodies, which showed an initial MFI >1000 and showed an MFI reduction ≥30% by treatment with fNP. For the HLA-A1 antigen, the MFI reduction for patient two is also given although it is <30%. For patient one, no whole blood was available. MFI= median fluorescence intensity, HLA= human leukocyte antigen.

***Supplementary table 4.*** *Effect of HLA-A1-fNP and HSA-fNP on reduced anti-HLA class II antibodies*

| **Anti-HLA-class II antibody** | **Relative MFI reduction (%)** | **Absolute MFI reduction** | **Absolute pre-treatment MFI** | **Absolute post-treatment MFI** |
| --- | --- | --- | --- | --- |
| **Treatment with HLA-A1 fNP** | | | | |
| Anti-HLA DRB1*04:01 antibody | 5 | 71 | 1525 | 1454 |
| Anti-HLA DRB1*04:02 antibody | 6 | 85 | 1401 | 1316 |
| Anti-HLA DRB1*04:03 antibody | 18 | 1141 | 6430 | 5289 |
| Anti-HLA DRB1*04:04 antibody | 6 | 87 | 1407 | 1320 |
| Anti-HLA DRB1*04:05 antibody | 13 | 152 | 1140 | 988 |
| Anti-HLA DRB1*07:01 antibody | 16 | 165 | 1035 | 870 |
| Anti-HLA DRB1*14:01 antibody | 15 | 156 | 1062 | 906 |
| **Treatment with HSA-fNP** | | | | |
| Anti-HLA DRB1*04:01 antibody | -5 | -69 | 1525 | 1594 |
| Anti-HLA DRB1*04:02 antibody | 2 | 30 | 1401 | 1371 |
| Anti-HLA DRB1*04:03 antibody | 10 | 654 | 6430 | 5776 |
| Anti-HLA DRB1*04:04 antibody | -7 | -100 | 1407 | 1507 |
| Anti-HLA DRB1*04:05 antibody | 7 | 77 | 1140 | 1063 |
| Anti-HLA DRB1*07:01 antibody | 7 | 75 | 1035 | 960 |
| Anti-HLA DRB1*14:01 antibody | 16 | 168 | 1062 | 894 |

***Supplementary table 4****. The absolute and relative MFI reduction of anti-HLA class II antibodies upon treatment of plasma from patient 1 with HLA-A1-fNP and HSA-fNP is shown as well as pre- and post-treatment MFI values. Only anti-HLA class II antibodies with an initial MFI >1000 and which were reduced by the HLA-A1-fNP treatment are shown. The “-” indicates an increase in MFI. MFI= median fluorescence intensity, HLA= human leukocyte antigen, HSA= human serum albumin, fNP= functionalized nanoparticle.*

**Supplementary figures**

***Supplementary figure 1.*** Patient flow chart

Patient flow chart. MFI = median fluorescence intensity, HLA = human leukocyte antigen.
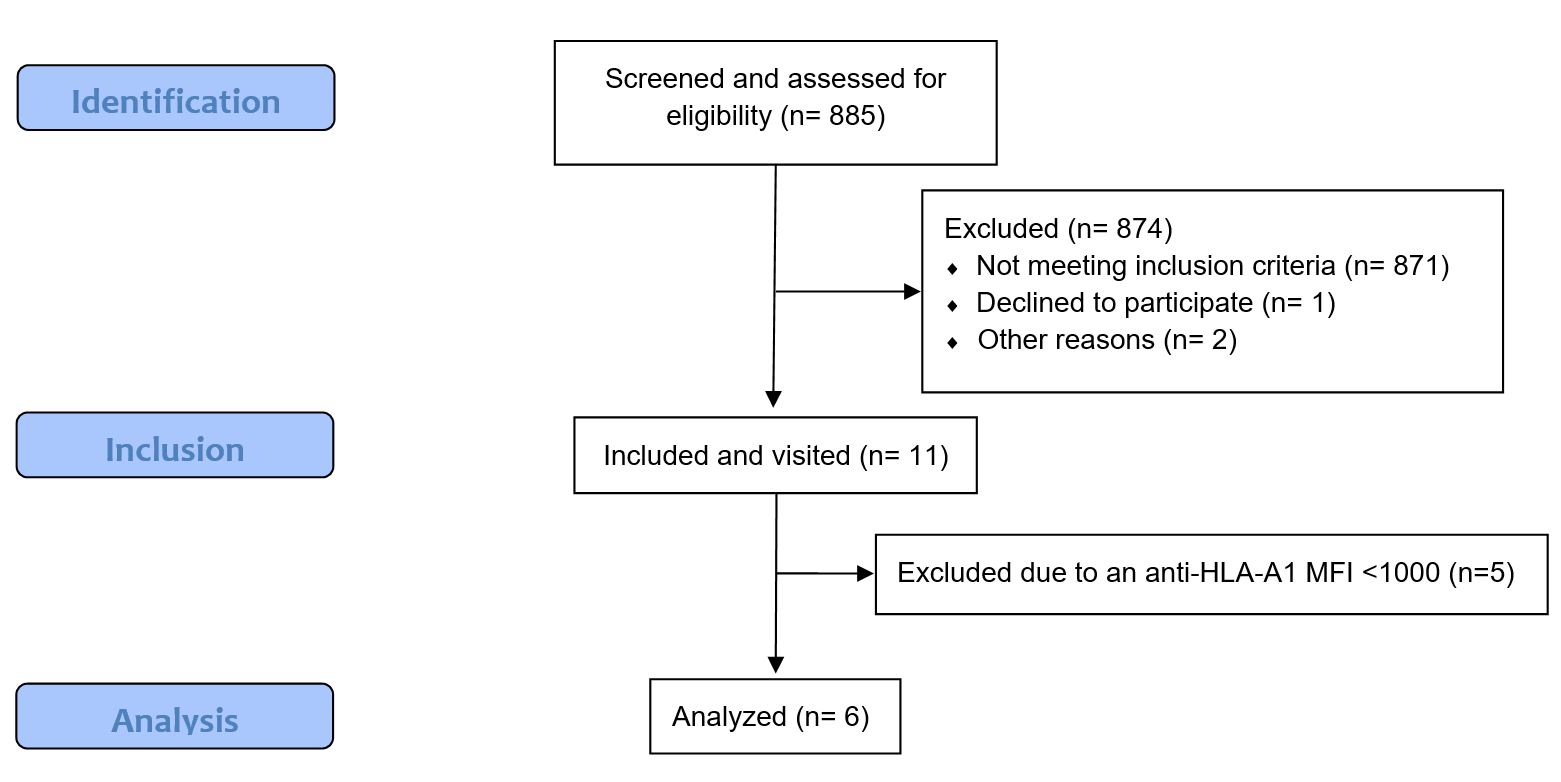


***Supplementary figure 2.*** Raw MFI values for the complete tested HLA class I panel in fNP treated plasma of patient 10

***
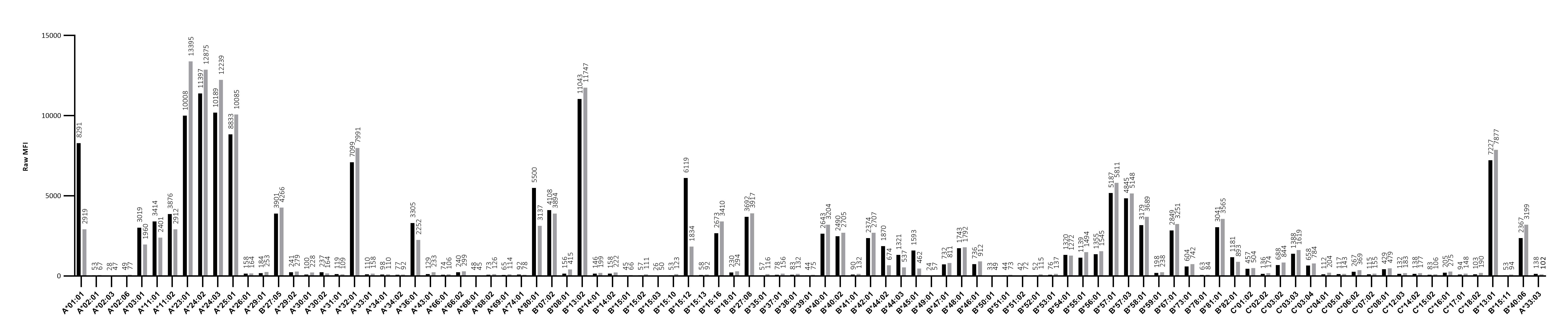
***

***Supplementary figure 3.*** Raw MFI values for the complete tested HLA class I panel in fNP treated whole blood of patient 10

**
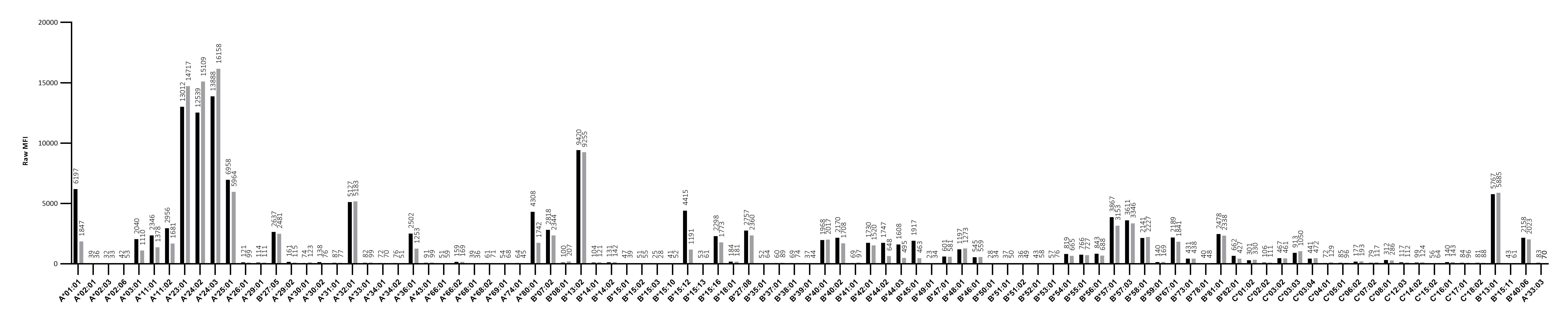
**

Exemplary graphical illustration of all tested HLA Class I antigens (pre and post fNP treatment) in patient 10. Raw MFI values are shown on the y-axis, and for each antigen (x-axis) above the column. The order of the antigens on the x-axis is according to the bead number of the LABScreen™ Single Antigen Beads HLA Class I panel. Raw MFI values prior to treatment with HLA-A1-fNP are shown in black, post-treatment values are shown in grey. ***Supplementary figure 2*** shows the result of HLA-A1-fNP treated plasma, ***supplementary figure 3*** the result of HLA-A1-fNP treated whole blood. MFI= median fluorescence intensity, HLA= human leukocyte antigen, fNP= functionalized nanoparticle.

***Supplementary figure 4.*** Device for testing fNP in flowing blood


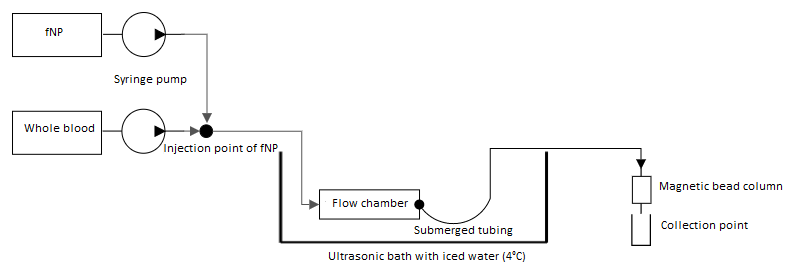


Schematic representation of the experimental setup to determine fNP uptake into PBMC under dynamic conditions (flow). The fNP-containing carrier solution was injected into anticoagulated whole blood. The tubing system was guided through a flow chamber and extension tubing, both submerged in an ultrasonic bath. fNP were removed from whole blood using a magnetic bead column. fNP = functionalized nanoparticles

**Experimental confirmation of mixing in the flowing system**

To ensure the mixing of the two streams (blood and nanoparticles), a preliminary experiment was performed. A pH indicator (phenolsulfophthalein, Sigma-Aldrich, St. Louis, MO, USA) was injected into NaOH (Sigma-Aldrich) as previously reported by Aubin et al. ^1^. Yellow phenolsulfophthalein became pink after mixing with (colourless) NaOH. Phenolsulfophthalein flow was 23 ml/h, NaOH flow 5.8 ml/min. Visual analysis indicates, mixing is fully complete after approximately 2 cm (**supplementary figure 5**).

| ***Supplementary figure 5.*** *Ensuring mixing in the device for testing fNP under flowing conditions* | |
| --- | --- |
| *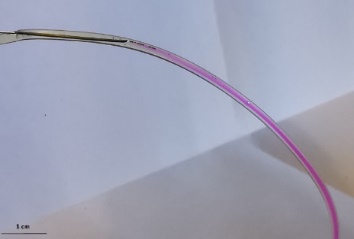* | *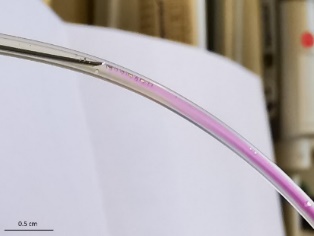* |
| *The yellow fluid (phenolsulfophthalein) flows at a rate of 23 ml/h and has a pH of 4.5. A cannula was used to inject NaOH (transparent, pH>14) at a flow rate of 5.8 ml/h. Upon mixing, a color change to pink was observed (indicated with a black arrow in the picture). Abbreviations: ml/h = milliliters per hour, NaOH = sodium hydroxide, cm = centimeters.* | |

**Literature Cited**

1 Aubin, J., Ferrando, M. & Jiricny, V. Current methods for characterising mixing and flow in microchannels. *Chemical Engineering Science* **65**, 2065–2093 (2010). <https://doi.org/10.1016/j.ces.2009.12.001>
